# New management grading for pig farms: management grading system using pig carcass weight, back fat thickness and k-means algorithm

**DOI:** 10.5713/ab.24.0350

**Published:** 2024-08-26

**Authors:** Youngho Lim, Jaeyoung Kim, Gwantae Kim, Jungseok Choi

**Affiliations:** 1 Department of Animal Science, Chungbuk National University, Cheongju 28644, Korea

**Keywords:** K-means, Landrace×Yorkshire×Duroc (LYD) Pig, Management Grade, Pig Grade, Regression Analysis, VCS2000

## Abstract

**Objective:**

This study categorized farm management levels to improve the productivity and uniformity of pork from pigs shipped from farms.

**Methods:**

A total of 48,298 pigs were grouped (A, B, C, D group) using the k-means algorithm, carcass weight and backfat thickness. The results of the grouping were used to classify Farm Management Grades (A, B, C, D grade).

**Results:**

The proportion of primal cuts in pigs, according to the new classification method, increased from group A to group D for shoulder blade, shoulder picnic, and ham, but decreased for loin and belly. In the regression analysis of the five primal cuts (shoulder blade, shoulder picnic, loin, belly, and ham) production (kg) for each group, all regression equations showed low errors (MAE<0.7), indicating that the model can predict the production of primal cuts by group. As the Farm Management Grade decreased, the proportion of pigs in the group with large differences from the mean of carcass weight and backfat thickness of the whole pig increased.

**Conclusion:**

The results of this study confirmed the differences in primal cut traits by pig grouping and created a method to classify farms who ship non-uniform pigs. This is expected to provide indicators for improvement and supplementation to farms that ship uneven pigs, helping to enhance the production of standardized pigs at the farm level.

## INTRODUCTION

Many countries around the world have their own grading standards for livestock, such as cattle and pigs, which often consider meat quantity characteristics. In the United States, the characteristics of beef cattle grades are considered through the retail cuts yield [1]. In Europe, the EUROP grid is commonly used to evaluate the quantity of meat based on external fat cover and carcass shape [2]. When evaluating the meat quantity of pigs, countries such as Korea and Japan consider the carcass weight and backfat thickness, and the European Union, Australia, the United States, and Germany consider lean meat percentage [3]. Thus, numerous countries employ a multitude of techniques to assess their livestock. However, most of the data utilized for grading was not utilized again. We hypothesized that, given that these data are continuously measured, they can be restructured and utilized for livestock management and agriculture.

Pigs are one of the most widely farmed animals in the world [4]. Pork is the most consumed meat in South Korea. Its consumption amount is about twice as much as beef and chicken [5]. The number of pigs in Republic of Korea in 2022 was 12% higher than in 2012 [6,7]. Pig consumption per capita has also increased from 19.2 kg in 2012 to 27.6 kg in 2021 [5,8]. Although pig production has increased, the number of pig farms has decreased from 6,000 in 2012 to 5,942 in 2021. Especially, the number of farms with less than 1,000 pigs has decreased from 3,080 in 2012 to 2,528 in 2021 [6,7]. This suggests that pig farming in South Korea is increasingly shifting toward larger-scale farming.

**Figure 1:**
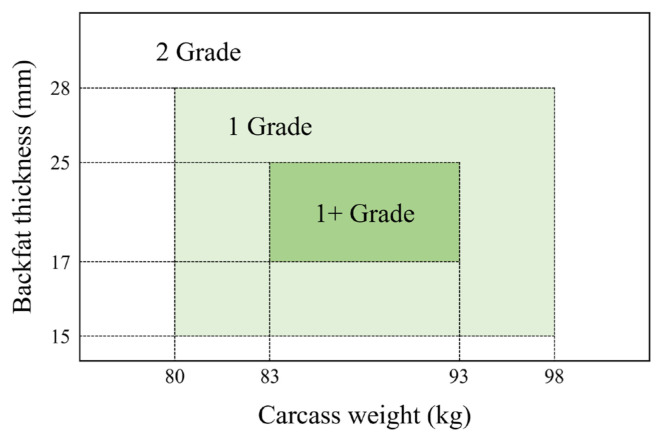
Meat quantity evaluation criteria in Korea’s pig grading system (based on scalding). The green color indicates the 1+ grade range, and the lime green color indicates the 1 grade range. The white color indicates the 2 grade range. The meat quantity evaluation was based on the following criteria: Grade 1+, 83 kg≤carcass weight<93 kg and 17 mm≤back fat thickness <25 mm; Grade 1, 80 kg≤carcass weight <98 kg and 15 mm ≤ back fat thickness <28 mm except the criteria of Grade 1+; Grade 2, all pig carcass except the criteria of Grade 1+ or Grade 1.

Pig breeding in South Korea aims at producing standard weight pigs [9–11]. This can be seen in the current Korean pork grading system. The Korean pig carcass grading system is divided into meat quantity evaluation and meat quality evaluation. The lower of the two determines the final grade of the pig [12]. Among them, meat quantity is evaluated by carcass weight and back fat thickness with three grades: 1+, 1, and 2 [12]. Pigs that do not qualify for the 1+ grade are judged as grade 1 and pigs that do not qualify for grade 1 are judged as grade 2 (Figure 1) [12]. All pigs raised in Korea are graded. Thus, the grade is known for all slaughtered pigs. Pigs that do not fit the standard are given a lower grade, which is economically less valuable.

Advanced livestock countries including Korea are managing economic pork production by promoting standards pig production at the national level by implementing their respective pig grading systems [3]. However, there is no management of standard pig productivity for farms that actually ship pigs on a national basis. Currently, the number of pig farms with less than 1,000 pigs is decreasing while the number of farms with more than 5,000 pigs is increasing, indicating that Korean pig farms are becoming larger [6], which can increase the influence of a single farm on pork productivity. Therefore, there is a need for an indicator that can present pork productivity at the farm level.

In addition, it is necessary to distinguish between differences in production of different cuts of pork. In the pork market, more desirable cuts are sold at higher prices [5,13]. There are opinions that the current pig grading system is ineffective because it does not guarantee the production or quality of highly preferred cuts such as pork belly and pork shoulder, even for pigs with high grades [14,15]. To address these complaints, there is a need for an indicator that can supplement the current grading system to show differences between grades. In this study, we used k-means, one of the unsupervised learning algorithms for pig discrimination, to group pigs and establish a management grade for farms using pig carcass weight and backfat thickness known to be the most important judgment indicators in the current Korean pig grading system. In addition, we tried to find out each primal cut production and prediction equation for each pig group through VCS2000 equipment to check the difference in meat characteristics by group.

Therefore, the objective of this study was to establish a manageable classification of pigs and farms using data measured during meat quantity grading of pig to improve pig productivity at the farm level and promote uniformity of pork produced. These classification criteria illustrate the potential for reconstructing the data utilized in grading and can be utilized as an important indicator for improving productivity by categorizing the level of specification management of pig farmers.

## MATERIALS AND METHODS

### Data

#### Animal and farm

Data of 48,298 pigs slaughtered at the Buyeong Livestock Auction Market (Juchon-myeon, Gimhae-si, Gyeongsangnam-do, Korea) from June 2022 to July 2022 were analyzed. Carcass weight and backfat thickness of slaughtered pigs were measured by mechanical grading according to detailed criteria for livestock product grading in Korea [12].

Farms analyzed in the study were those that shipped pigs to the Buyeong Livestock Auction Market from June 2022 to July 2022, Farms with less than 30 pigs were excluded (total number of farms = 94). Data for each farm’s shipped pigs were pigs shipped and slaughtered at the Bukyeong Livestock Auction Market from June 2022 to July 2022.

#### VCS2000

A VCS2000 (E+V Technology GmbH, Oranienburg, Germany) is an automatic pork carcass measuring machine that consists of two color cameras and one monochromatic camera, a background device, a lighting device, a carcass holder, a carcass guide, a vision program, a computer, and spare parts [16]. The VCS2000 is installed and used during the pig slaughtering process. It can capture images of the bisected pig carcass with two color cameras and one monochromatic camera. Based on these images, it can predict about 52 characteristics related to the pig carcass [17–19]. These include production of primal cuts, fat percentage, and so on. In this experiment, we used the production of five primal cuts (shoulder blade, shoulder picnic, loin, belly, ham) measured by VCS2000.

### K-mean modeling

#### Data standardization

The algorithm used for pig grouping and farm management class model is k-means, which is a vector-dependent algorithm. Therefore, carcass weight and backfat thickness of pigs (48,298) used for k-means modeling were standardized by absolute (abs) z-score. Abs z-score is the absolute value of the z-score (standard score), which is the data (xi) minus the mean (μ) of the data population divided by the standard deviation (σ) of the data population. The expression for the absolute (abs) z-score used for data standardization is shown in Eq. (1).


Eq. (1) $$\left|\frac{x^{i}-\mu }{\sigma }\right|$$

#### K-means clustering algorithm

K-means clustering algorithm is a kind of vector quantization method that divides n objects (data) into k clusters so that each object (data) belongs to the closest cluster [20,21]. Therefore, objects belonging to the same cluster are similar to each other but not similar to objects belonging to other clusters [21]. The k-means model used was set to have a total of nine clusters. To set initial cluster centers, we used the ‘k-means++’ method, which could select cluster centers based on the distance between data points. The ‘k-means++’ algorithm first sets one centroid (c(1)) randomly from the dataset. It then sets the i-th data point (x(i)) as a new centroid with the probability given by Eq. (2). This is repeated until k centroids are set [22]. D(x(i)) is the distance of x(i) to the nearest centroid already selected.


Eq. (2) $$D{\left(x^{\left(i\right)}\right)}^{2}/\sum_{j=1}^{m}D{\left(x^{\left(j\right)}\right)}^{2}$$

**Figure 2:**
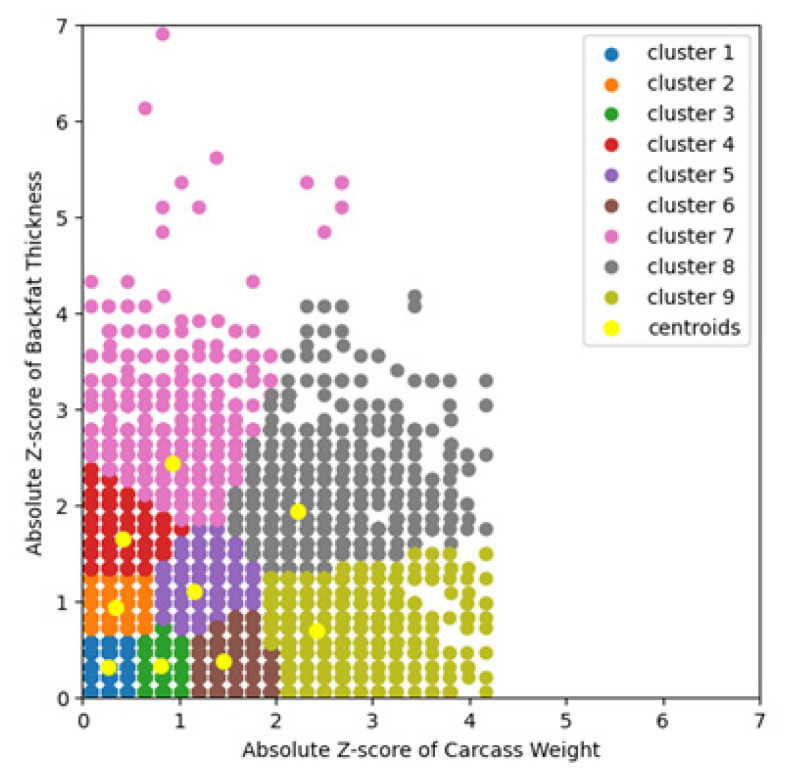
K-means results of standardized data with Absolute Z-score (number of clusters = 9). The number of pigs used in the study was 48,298, slaughtered in June and July. Each dot represents a data point (pig carcass), color-coded according to its cluster. The clusters to which each colored data point corresponds are as follows: blue (cluster 1), data points for cluster 1; orange (cluster 2), data points for cluster 2; green (cluster 3), data points for cluster 3; red (cluster 4), data points for cluster 4; purple (cluster 5), data points for cluster 5; brown (cluster 6), data points for cluster 6; pink (cluster 7), data points for cluster 7; grey (cluster 8), data points for cluster 8; olive (cluster 9), data points for cluster 9. Clusters are determined using k-means clustering. The centroids of each cluster are indicated by yellow dots.

The number of clusters in the k-means model used in this study was set to 9, with 200 initializations for the initial cluster centers and a maximum of 1,000 iterations for the process of moving cluster centers (Figure 2).

#### Pig grouping

The nine clusters generated by the k-means model were grouped into group I, group II, group III, and group IV (group I, cluster 1; group II, cluster 2 and cluster 3; group III, cluster 4, cluster 5, and cluster 6; group IV, cluster 7, cluster 8, and cluster 9) (Table 1).

#### Farm management grade

Farm Management Grade was categorized into A grade, B grade, C grade, and D grade. The classification method was as follows: i) calculating the average carcass weight and backfat thickness of all pigs in the farm; ii) scaling by abs z-score (Eq.(1)) using the mean and standard deviation of the carcass weight and backfat thickness of all pigs (Supplementary Table S1); and iii) the grade obtained when put into the k-means model of this study (Figure 2) was the Farm Management Grade of the farm.

### Statistical analysis

One-way analysis of variance and Bonferroni Test were conducted to compare carcass weight, backfat thickness, and production of five primal cuts (shoulder blade, shoulder picnic, loin, belly, and ham) by pig grouping. To obtain regression equations for each group, carcass weight and backfat thickness were set as independent variables and the production of primal cuts was set as the dependent variable. The accuracy of the regression equation was measured by the coefficient of determination R^2^ and the error rate was measured by the mean absolute error (MAE).

### Software

All analyses and modeling in the study were conducted in the Python 3.11 environment. Pandas [23] version 2.1.1 and Numpy [24] version 1.26.0 were used for data processing and analysis, Scikit-learn [25] version 1.3.0 was used for data normalization and k-means modeling. Scipy [26] version 1.11.2 and Statsmodels [27] version 0.14.0 were used for statistical analysis, Bonferroni test, and regression analysis.

## RESULTS

### K-means model and pig grouping

Pigs were grouped using the k-means algorithm as a process to create the Farm Management Grade (Table 1, Figure 2). Pigs with larger values of abs z-score of carcass weight were those with larger differences from the average carcass weight of all pigs. Similarly, pigs with larger values of abs z-score of backfat thickness were those with larger differences from the average backfat thickness of all pigs. Therefore, pigs in cluster 1 were those with the least variation in the average carcass weight and average backfat thickness of all pigs. As the cluster number increased, pigs in that cluster had more variations in average carcass weight, average backfat thickness, or both. Group appearance rate of pigs in this study was 24.9% in group I, 33.3% in group II, 31.5% in group III, and 10.3% in group IV (Table 1).

### Comparison of pig grouping and the current Korean pig grading system

To compare groups categorized by abs z-score of carcass weight and backfat thickness of pigs with the current Korean pig grading system using carcass weight and backfat thickness, actual grades of pigs in each group (I, II, III, and IV) were examined and compared (Table 2). When we examined the first grade of pigs in each group, we found that all pigs in group I were graded 1+. Pigs in group II were all grades 1+ and 1, with a predominance of 1+ grade. Pigs in group III had the highest proportion of grade 1, with grade 2 being the second most common. In group IV, no pigs were rated 1+ and most pigs were rated 2. Examining final grades of pigs in each group, we found that group I was dominated by pigs graded 1+, while group II had a similar representation for grades of 1+ and 1. Group III had the highest percentage of pigs graded 1, with the second highest percentage of pigs graded 2. Group IV had no pigs graded 1+ It was dominated by pigs graded 2. The distribution of first and final grades for pigs in each group was very similar.

### Meat quantity characteristics by pig group

To determine differences in carcass traits and meat quantity characteristics between groups, we compared carcass weight, backfat thickness, and production ratio of the five primal cuts by groups (Table 3). Pigs in group I had higher carcass weight and backfat thickness than pigs in the other groups (groups II, III, and IV) (p<0.05). Pigs in group I had higher carcass weight than pigs in group II. Pigs in groups I and III had thinner backfat thickness than pigs in group II (p<0.05). For production ratio of primal cuts, pigs in group IV had lower shoulder blade and shoulder picnic ratio than pigs in other groups (I, II, and III). Pigs in group III had lower shoulder blade ratio than pigs in groups I and II (p<0.05). In contrast, the production ratio of loin and belly was higher for pigs in group IV than for pigs in other groups (I, II, and III). Pigs in group I had a lower loin production ratio than pigs in other groups (II, III, and IV) (p<0.05). Also, pigs in group I had a lower belly production rate than pigs in group II (p<0.05). Ham production ratio was higher for pigs in groups I, II, III, and IV (p<0.05). Standard deviations of carcass weight, backfat thickness, and the five major carcass parts increased from group I to group IV.

### Regression of primal cuts production by pig group

To compensate for the fact that the production of primal cuts is not known through the current Korean pig grading system, we conducted a regression analysis for the prediction of primal cuts by setting primal cuts as the dependent variable and carcass weight and backfat thickness as independent variables (Table 4; Supplementary Figure S1). Regression equations for each group of five primal cuts of pigs are shown in Table 4. Graphs of regression models of primal cuts, carcass weight, and backfat thickness for each group are shown in Supplementary Figure S1. For all primal cuts, R^2^ was low for the regression equations in order of groups I, II, III, and IV (Table 4). However, MAE was not highly different between groups. It was lower in the following order: groups I, II, III, and IV (Table 4).

### Farm Management Grade

Farm Management Grades were created to increase the productivity of farms and the uniformity of pigs raised. The number of farms in each management grade is shown in Table 5, with the highest number of farms in grade B and the lowest number of farms in grades A and D (Table 5).

### Comparing Farm Management Grade and pig group

To compare the proportion of pig groups belonging to each farm according to the Farm Management Grade, we checked the prevalence of pigs belonging to each group according to the Farm Management Grade (Table 6). A grade is the highest percentage of pigs belonging to group I, B grade is the highest percentage of pigs belonging to group II, C grade is the highest percentage of pigs belonging to group III, and D grade is the highest percentage of pigs belonging to group IV (Table 6). As the Farm Management Grade increased from A to D, the proportion of pigs belonging to the group IV increased (Table 6).

### Comparison of Farm Management Grade and the current Korean pig grading system

For comparing Korean pig grade appearance rate by Farm Management Grade, we identified the current Korean pig grade appearance rate for pigs in farms according to the Farm Management Grade (Table 6). The mean of the current grade appearance rate for pigs in each farm according to the Farm Management Grade is shown in Table 7. As the Farm Management Grade of the farm increased from A to D, the proportion of pigs in Grade 1+ decreased while the proportion of pigs in Grade 2 increased (Table 6). The proportion of pigs in grade 1 increased from grade A to grade C, but decreased in grade D (Table 6).

## DISCUSSION

### Current Korean pig grading system

More than 99% of pigs in Korea are slaughtered by scalding [28]. According to the meat grading system for scalding carcasses, the criteria for grade 1+ are 83 kg≤carcass weight<93 kg and 17 mm≤back fat thickness<25 mm (Figure 1). Grade 1 is for 80 kg≤carcass weight<98 kg and 15 mm≤back fat thickness <28 mm, except for pig carcasses that qualify for Grade 1+ (Figure 1). Grade 2 shows all pig carcass that do not match the criteria of Grade 1+ or Grade 1 (Figure 1) [12]. This shows that the current Korean pig meat grading system is centered on the production of standard weight pigs. In addition, the grading of pigs is divided into two parts: the first grade, which is the meat quantity grade, and the second grade, which is the meat quality grade that consists of appearance, meat quality, and defect points. The lower of the two results is the final grade of the pig [12]. However, as of 2018, the proportion of pigs graded down due to secondary grading was 5.2% [29]. In our study, the proportion of first grade and final grade did not show a big difference (Table 2). This suggests that in the current Korean pig grading system, carcass weight and backfat thickness, which are used in the meat quantity grade (first grade), have a greater impact than meat quality grade (second grade), which is measured by appearance, meat quality, and defect items.

### Pig grouping and the current Korean pig grading system

As a first step to establish a Farm Management Grade, which is expected to help increase the uniformity and productivity of pigs at the farm level, we used the k-means algorithm to group pigs using carcass weight and backfat thickness and compared it with the current pig grading system. The k-means algorithm used in this study is an effective clustering algorithm that has been used in various research fields for various purposes such as data grouping, data classification, identification, data preprocessing, and data mining [20,22,30–33].

The graded prevalence of pigs in South Korea in 2022 as reported by KAPE [34] was 33.9% for grade 1+, 33.7% for grade 1, and 28.3% for grade 2. In this study, the proportion of pigs in each group was 25% for group I, 33.3% for group II, 31.5% for group III, and 10.3% for group IV (Table 1). These results show that the proportion of pigs in each group is not significantly different from the prevalence of grading, but a more granular distribution. In addition, farmers can check carcass weight and backfat thickness of each pig to see which cluster or group their pigs or the representative value of their farm (such as the average or the value used in our proposed Farm Management Grade) belongs to and which part of the carcass weight and backfat thickness deviates from the standard weight pig in Korea.

Currently, the Korean pig grading system is geared toward the production of standard pigs. It determines meat quantity grade through carcass weight and backfat thickness. Average carcass weight and backfat thickness of pigs were in the 1+ grade range or close to the 1+ grade range (Figure 1). Variables used in k-means in this study were carcass weight and backfat thickness standardized and scaled to absolute values (Eq. 1). Averages of carcass weight and backfat thickness of pigs used in this study corresponded to a 1+ grade according to the current pig grading system (Supplementary Table S1). These values were used for scaling. Therefore, pigs belonging to clusters with centroids, smaller abs z-score of carcass weights, and abs z-score of backfat thickness were more likely to be graded 1+, while clusters with centroids and larger abs z-score of carcass weight and abs z-score of backfat thickness were more likely to have pigs with lower grading results (Table 2; Supplementary Table S3).

### Meat quantity characteristics of pigs by group

One of the consumer complaints about the current Korean pig grading system is that grades do not distinguish production by cut [15]. To help with this problem, this study compared carcass weight, backfat thickness, and production of five primal cuts according to pig groups created by the k-means model and created regression equations to predict production of each cut.

Variations in carcass weight and backfat thickness increased from group A to group D because variables used to form clusters were scaled by absolute values of z-score values of carcass weight and backfat thickness (Table 3). In Korean pork grades, the lower the grade, the larger the deviations of carcass weight and backfat thickness, which had the same trend as results of this experiment (Figure 1, Table 3). Deviations of carcass weight and backfat thickness, which evaluates meat quantity of pigs from group I to group IV, affected deviations of five primal cuts (Table 3). In a study comparing the relationship between the Korean pig grading system and Landrace×Yorkshire×Duroc (LYD) pig primal cuts [35], the higher the pig grade (closer to 1+ grade), the lower the belly production and the higher the production of shoulder blade, shoulder picnic, ham, and loin. As pig groups modeled by k-means in this study changed from I to IV, the dominant grade of pigs in each group (LYD pigs) changed from 1+ to 1 and then to 2 (Table 2). In this study, except for the loin, the proportion of belly decreased from group I to group IV, while proportions of shoulder blade, shoulder picnic, and ham increased. These results were similar to those of Park et al [35] after investigating the production of pork primal cuts by grade in Korea. Therefore, the proportion of pork primal cuts by group tends to be similar to that of Korean grades, which is likely due to the fact that both grades are categorized based on carcass weight and backfat thickness (Table 2).

Regression analyses were performed to predict the weight of each primal cut per grade, with carcass weight and backfat thickness per group as independent variables and the production of each primal cut as the dependent variable. Evaluation metrics of the regression analysis were R^2^ indicating the explanatory power of the regression equation and MAE indicating the error between predicted and actual values [36,37]. R^2^ is ultimately determined by reflecting the mean of the y-values of the data (in this case, the production of each batch of meat) [36]. Even if the groups have similarly sized residuals, the farther the data are from the mean of the y-values, the larger the sum of squares regression (SSR) and the higher the R^2^, while the closer the data are to the mean of the y-values, the smaller the SSR and the lower the R^2^. Thus, the reason why groups I, II, III, and IV had similar MAEs but large differences in R^2^ is that the data became more distant from the mean of each group as they progressed from I to IV (Supplementary Figure S1). Therefore, it was concluded that the regression equation was able to predict the lean meat yield of each pig. Results of this study can be used to support the current pig grading system by providing information and predicting the weight of each primal cut of a pig.

### Farm Management Grade

As of 2022, Grade 1+ and Grade 1 each accounted for about one-third of all pigs shipped in the Republic of Korea [34]. Therefore, the Farm Management Grade, which was obtained using average values of carcass weight and backfat thickness of each farm, expected that the number of farms in Grade A and Grade B would be about 30% each. However, actual results showed that 66.67% of farms were class B, 31.18% were class C, and only 1% were classes A and D, respectively. This is probably due to a difference between the Korean pig grading system and our pig grouping criteria. The Farm Management Grade of a farm is determined according to the corresponding cluster by inputting mean carcass weight and mean backfat thickness of pigs shipped from a farm into the k-means model described in this study. The grouping of pigs according to the k-means model in this study is more granular than the distribution of pigs according to current Korean pig grades (Tables 1, 2). This can also be confirmed by comparing the proportion of pig groups according to Farm Management Grade with the proportion of Korean pig grades (Tables 6, 7). According to pig grouping, pigs that fell under the 1+ grade of the Korean pig grading system were mostly divided into groups I and II (Table 2). This had a significant impact on the number of pigs in the I group, which was mostly composed of pigs having the 1+ grade (Korean pig grading), being smaller than the number of pigs having the 1+ grade (Tables 1, 2). This had a significant impact on the number of A-grade farms with mainstream pigs in the I group. In addition, pigs in group II accounted for the largest proportion (33.3%), followed by pigs in group III (31.5%). These results led to the fact that the number of farms falling under the B grade was the largest. Most of the farms fell under B and C grade among Farm Management Grades.

## CONCLUSION

We grouped pigs according to carcass weight and backfat and presented a Farm Management Grade. The new grouping of pigs in this study can predict primal cut production with a small error using only carcass weight and backfat thickness. This could assist the current Korea pig grading system by providing predicted weights per cut based on carcass traits measured during meat quantity grading. The Farm Management Grade can be used to identify differences in pigs shipped from each farm, making it easier for pig farmers to find out which parts of the carcass weight and backfat thickness are different from standard weight pigs. Accordingly, it is believed that more standard pigs can be produced through methods such as selective shipping or feeding finishing-stage feed based on results of Farm Management Grade. Therefore, Farm Management Grade can be used as an indicator for specification management and breeding direction at the farm level.

## Figures and Tables

**Table 1:** Cluster number and number of pigs in each group

Group	Cluster number^1)^	Number of pigs (n, %)
I	Cluster 1	12,026 (24.9)
II	Cluster 2	8,309 (17.2)
	Cluster 3	7,778 (16.^1)^
III	Cluster 4	4,525 (9.4)
	Cluster 5	6,081 (12.6)
	Cluster 6	4,594 (9.5)
IV	Cluster 7	1,610 (3.3)
	Cluster 8	1,455 (3.0)
	Cluster 9	1,920 (4.0)

The number of pigs used in the study was 48,298, slaughtered in June and July.

1) Each cluster is shown in Figure 1.

**Table 2:** Percentage of pigs in each Korean pig grade for each group

Group^2)^	Grade	Korean pig grade^1)^	

		Appearance rate of 1st grade (%)	Appearance rate of final grade (%)
I	1+	100	95
	1	-	5
	2	-	-
II	1+	55	50
	1	45	49
	2	-	1
III	1+	8	7
	1	57	57
	2	35	36
IV	1+	-	-
	1	7	7
	2	93	93

The number of pigs used in the study was 48,298, slaughtered in June and July.

1) Korean pig grades (final grade) were determined by the lower of the two grades, based on the quantity of the carcass (1st grade), and the appearance and quality of the carcass (2nd grade). 1+ is the highest grade, followed by grades 1 and 2.

2) Group I, pigs in cluster 1; Group II, pigs in clusters 2 and 3; Group III, pigs in clusters 4,5,6; Group IV, pigs in clusters 7, 8, 9 (Table 1, Figure 2).

**Table 3:** Comparison of carcass weight, backfat thickness, and 5 primal cuts production rates in each group

Treatment		Group^1)^			

		I	II	III	IV
Carcass weight (kg)		86.43±1.66^b^	86.32±3.45^c^	86.34±6.13^bc^	87.90±10.99^a^
Backfat thickness (mm)		21.99±1.37^c^	22.33±2.83^b^	21.98±4.58^c^	23.16±7.09^a^
Primal cuts (%)^2)^	Shoulder blade	6.65±0.25^a^	6.65±0.25^a^	6.64±0.26^b^	6.59±0.27^c^
	Shoulder picnic	12.93±0.35^a^	12.93±0.38^a^	12.93±0.40^a^	12.89±0.45^b^
	Loin	11.53±0.34^c^	11.56±0.38^b^	11.55±0.44^b^	11.63±0.54^a^
	Belly	19.06±0.86^c^	19.10±0.91^b^	19.09±0.99^bc^	19.23±1.14^a^
	Ham	22.02±0.81^a^	21.99±0.83^b^	21.93±0.87^c^	21.80±0.94^d^

The number of pigs used in the study was 48,298, slaughtered in June and July.

1) Group I, pigs in cluster 1; Group II, pigs in clusters 2 and 3; Group III, pigs in clusters 4, 5, 6; Group IV, pigs in clusters 7, 8, 9 (Table 1, Figure 2).

2) The production ratio of each primal cut of the pig was obtained as follows: (primal cut weight/carcass weight)×100. The number of pigs used in the study was 48,298.

a–d Values in the same row with different superscripts denote a statistically significant difference, determined by their means±standard deviations (p<0.05).

**Table 4:** Regression equations for five primal cuts weight in groups

Dependent variable	Intercept (β0)	Regression coefficient of carcass weight (β1)	Regression coefficient of Backfat thickness (β2)	Mean absolute error	R^2^
Group I					
Shoulder blade	0.7503	0.0586	−0.0033	0.16	0.173
Shoulder picnic	−0.6278	0.1438	−0.0283	0.23	0.385
Loin	−1.1656	0.1156	0.0520	0.23	0.348
Belly	−3.3129	0.2122	0.0657	0.58	0.200
Ham	0.2182	0.2188	−0.0044	0.51	0.210
Group II					
Shoulder blade	0.4320	0.0607	0.0030	0.16	0.488
Shoulder picnic	−0.6909	0.1427	−0.0207	0.24	0.702
Loin	−0.9945	0.1141	0.0500	0.23	0.684
Belly	−3.1669	0.2098	0.0696	0.59	0.506
Ham	0.5907	0.2160	−0.0115	0.52	0.518
Group III					
Shoulder blade	0.4396	0.0601	0.0046	0.17	0.896
Shoulder picnic	−0.6446	0.1411	−0.0168	0.25	0.864
Loin	−1.0174	0.1155	0.0466	0.24	0.865
Belly	−3.2610	0.2108	0.0708	0.59	0.776
Ham	0.5883	0.2151	−0.0105	0.53	0.757
Group IV					
Shoulder blade	0.2991	0.0609	0.0060	0.18	0.896
Shoulder picnic	−0.7132	0.1407	−0.0137	0.27	0.940
Loin	−0.9785	0.1164	0.0430	0.28	0.941
Belly	−3.0578	0.2111	0.0633	0.63	0.909
Ham	0.2460	0.2178	−0.0103	0.58	0.897

The pigs used in the study were slaughtered in June and July.

The confidence intervals for each regression are shown in Supplementary Table S2.1–2.4.

The form of the regression expression is as follows: y = β1×1 (carcass weight) + β2×2 (backfat thickness) + β0.

**Table 5:** Number and percentage of farms by farm management grade

Item	A grade	B grade	C grade	D grade
Farm (n = 93)	1 (1.08%)	62 (66.67%)	29 (31.18%)	1 (1.08%)

Total number of farms: 93.

Farm Management Grade is the management grade obtained when the mean values of carcass weight and backfat thickness of pigs in each farm were standardized to the mean and standard deviation of all pigs (Supplementary Table S1) and entered a k-means model (Figure 2). These results exclude farms with fewer than 25 pigs shipped.

**Table 6:** Percentage of pig group by Farm Management Grade

Pig group^1)^	Farm Management Grade			

	A grade	B grade	C grade	D grade
I (%)	47.24	28.27±5.91	17.72±3.75	4.3
II (%)	31.5	35.52±3.52	28.66±3.08	6.92
III (%)	20.47	29.84±5.16	36.55±4.45	28.16
IV (%)	0.79	6.40±3.26	17.0±4.31	60.62

Total number of farms: 93.

The number of pigs used in the study was 48,298, slaughtered in June and July.

Farm Management Grade is the management grade obtained when the mean values of carcass weight and backfat thickness of pigs in each farm were standardized to the mean and standard deviation of all pigs (Supplementary Table S1) and entered a k-means model (Figure 2).

There is one farm in each of the A and D grades.

These results exclude farms with fewer than 25 pigs shipped.

1) Group I, pigs in cluster 1; Group II, pigs in clusters 2 and 3; Group III, pigs in clusters 4, 5, 6; Group IV, pigs in clusters 7, 8, 9 (Table 1, Figure 2).

**Table 7:** Appearance rate of Korean pig grade by Farm Management Grade

Korean pig grade (Final grade)^1)^	Farm Management Grade			

	A grade	B grade	C grade	D grade
1+ grade (%)	61.42	47.88±8.00	32.04±7.47	4.77
1 grade (%)	30.71	35.33±5.22	37.55±5.15	13.13
2 grade (%)	7.87	16.79±5.01	30.40±7.24	82.10

Total number of farms: 93.

The number of pigs used in the study was 48,298, slaughtered in June and July.

Farm management grade is the management grade obtained when the mean values of carcass weight and backfat thickness of pigs in each farm were standardized to the mean and standard deviation of all pigs (Supplementary Table S1) and entered a k-means model (Figure 2).

There is one farm in each of the A and D grades.

These results exclude farms with fewer than 25 pigs shipped.

1) Korean pig grades (final grade) were determined by the lower of the two grades, based on the quantity of the carcass (1st grade), and the appearance and quality of the carcass (2nd grade). 1+ is the highest grade, followed by grades 1 and 2.

## SUPPLEMENTARY MATERIAL

**Supplementary Figure S1.1.** Primal cut production (kg) as a function of carcass weight (kg) and backfat thickness (mm) for pigs in group. **Supplementary Figure S1.2.** Primal cut production (kg) as a function of carcass weight (kg) and backfat thickness (mm) for pigs in group. **Supplementary Figure S1.3.** Primal cut production (kg) as a function of carcass weight (kg) and backfat thickness (mm) for pigs in group. **Supplementary Figure S1.4.** Primal cut production (kg) as a function of carcass weight (kg) and backfat thickness (mm) for pigs in group.

**Supplementary Table S1.** Mean and standard deviation of carcass weight and backfat thickness for pigs used in the study **Supplementary Table S2.1.** Confidence intervals for regression equations for five primal cuts of pigs in group **Supplementary Table S2.2.** Confidence intervals for regression equations for five primal cuts of pigs in group **Supplementary Table S2.3.** Confidence intervals for regression equations for five primal cuts of pigs in group **Supplementary Table S2.4.** Confidence intervals for regression equations for five primal cuts of pigs in group **Supplementary Table S3.** Centroid coordinates for each cluster **Supplementary Table S4.** Regression equations for five primal cuts weight in whole data **Supplementary Table S5.** Confidence intervals for regression equations for five primal cuts of whole data
