# Supplementary material for: New management grading for pig farms: management grading system using pig carcass weight, back fat thickness and k-means algorithm
Source: Anim Biosci. 2024 Aug 26;38(2):371–80. doi: 10.5713/ab.24.0350 (PMC11725751; doi:10.5713/ab.24.0350)
Supplement: Supplementary file 1 [file ab-24-0350-Supplementary-Fig.pdf]

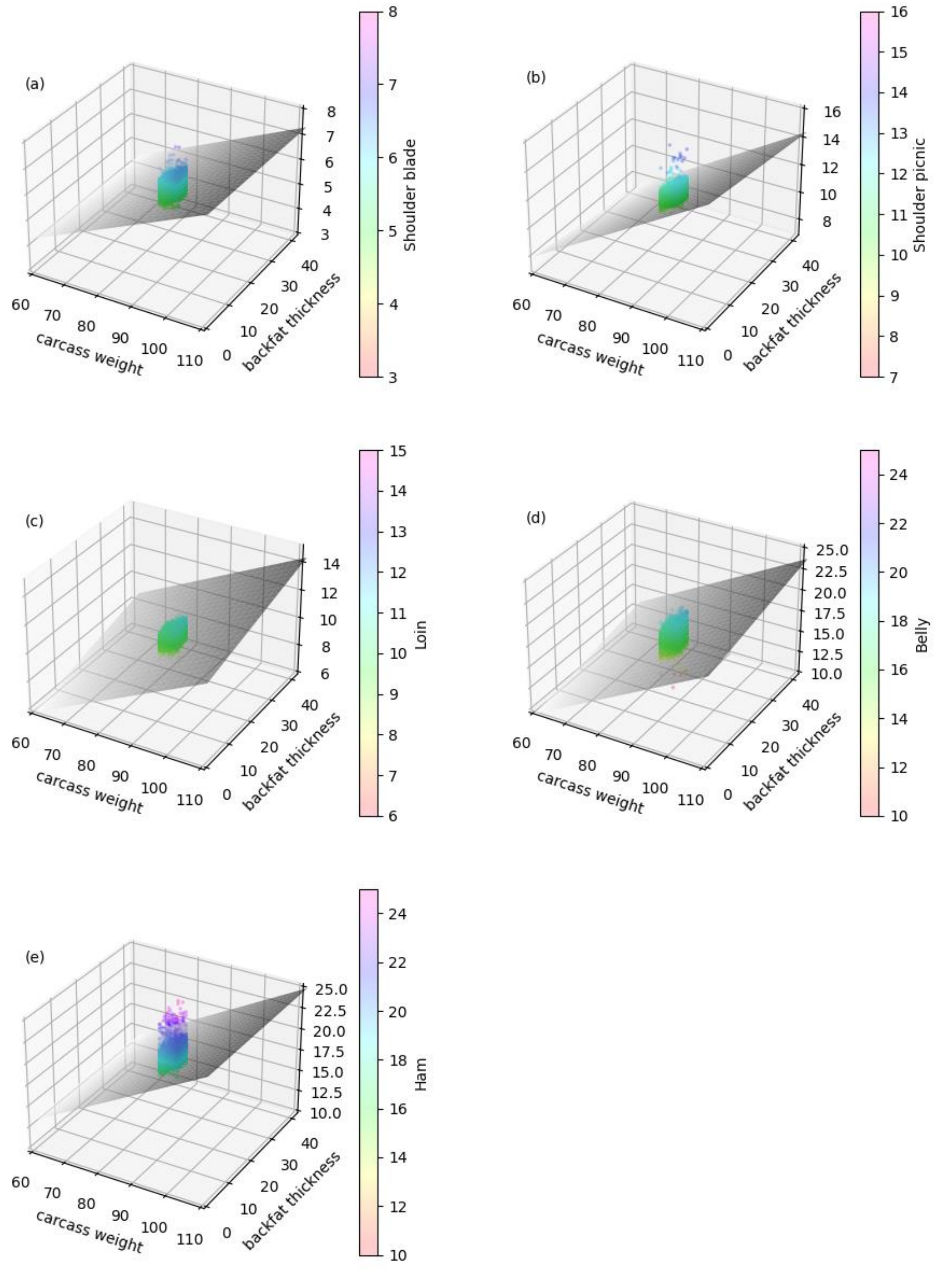

Supplementary Figure 1.1. Primal cut production (kg) as a function of carcass weight (kg) and backfat thickness (mm) for pigs in group I. The primal cut production in each graph represents (a) shoulder blade, (b) shoulder picnic, (c) loin, (d) belly, and (e) ham. The black plots in each graph are the graphs of the regression models from Table 4.1 with carcass weight and backfat thickness as independent variables and each primal cut production as the dependent variable.

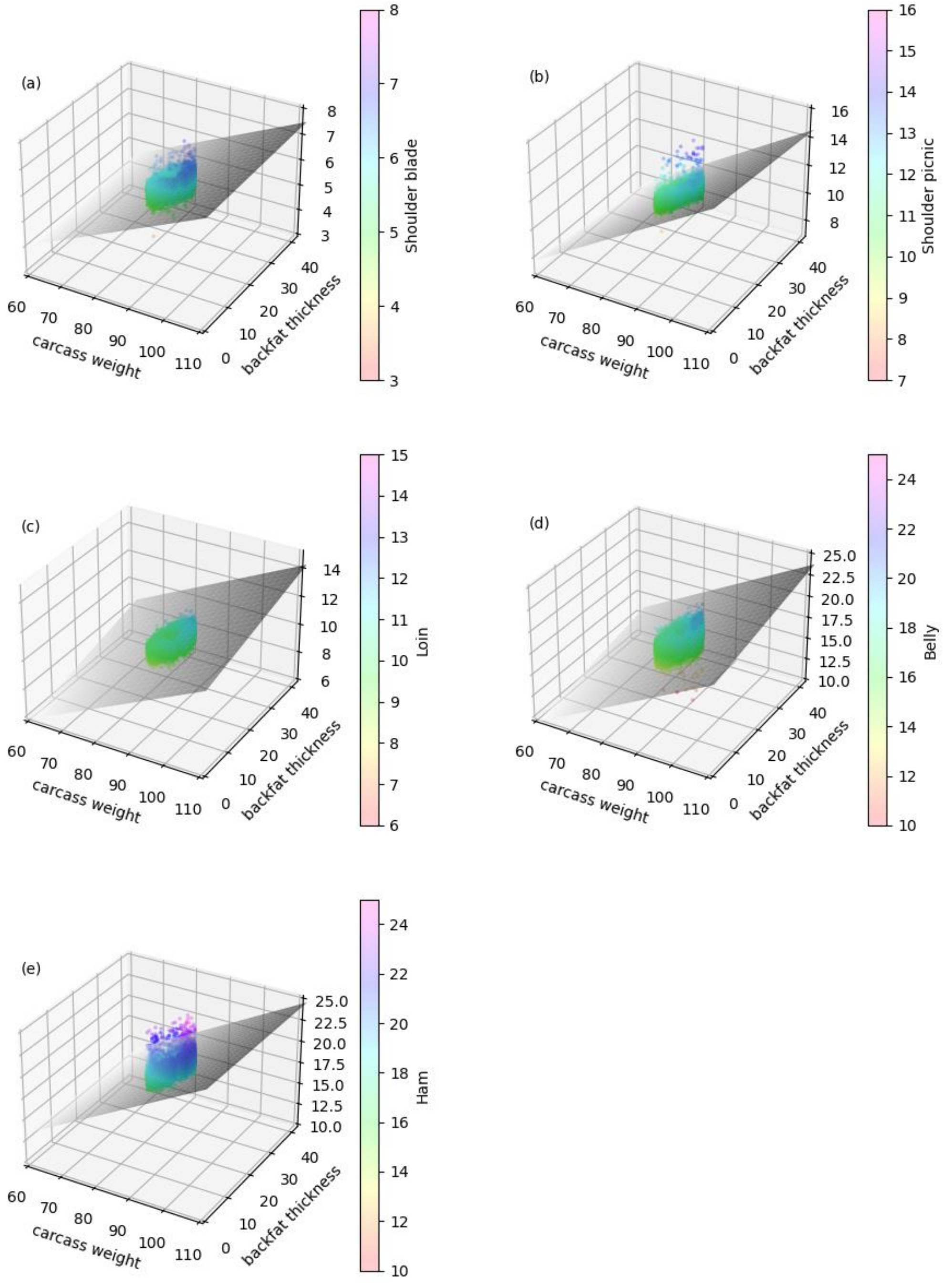

Supplementary Figure 1.2. Primal cut production (kg) as a function of carcass weight (kg) and backfat thickness (mm) for pigs in group II. The primal cut production in each graph represents (a) shoulder blade, (b) shoulder picnic, (c) loin, (d) belly, and (e) ham. The black plots in each graph are the graphs of the regression models from Table 4.2 with carcass weight and backfat thickness as independent variables and each primal cut production as the dependent variable.

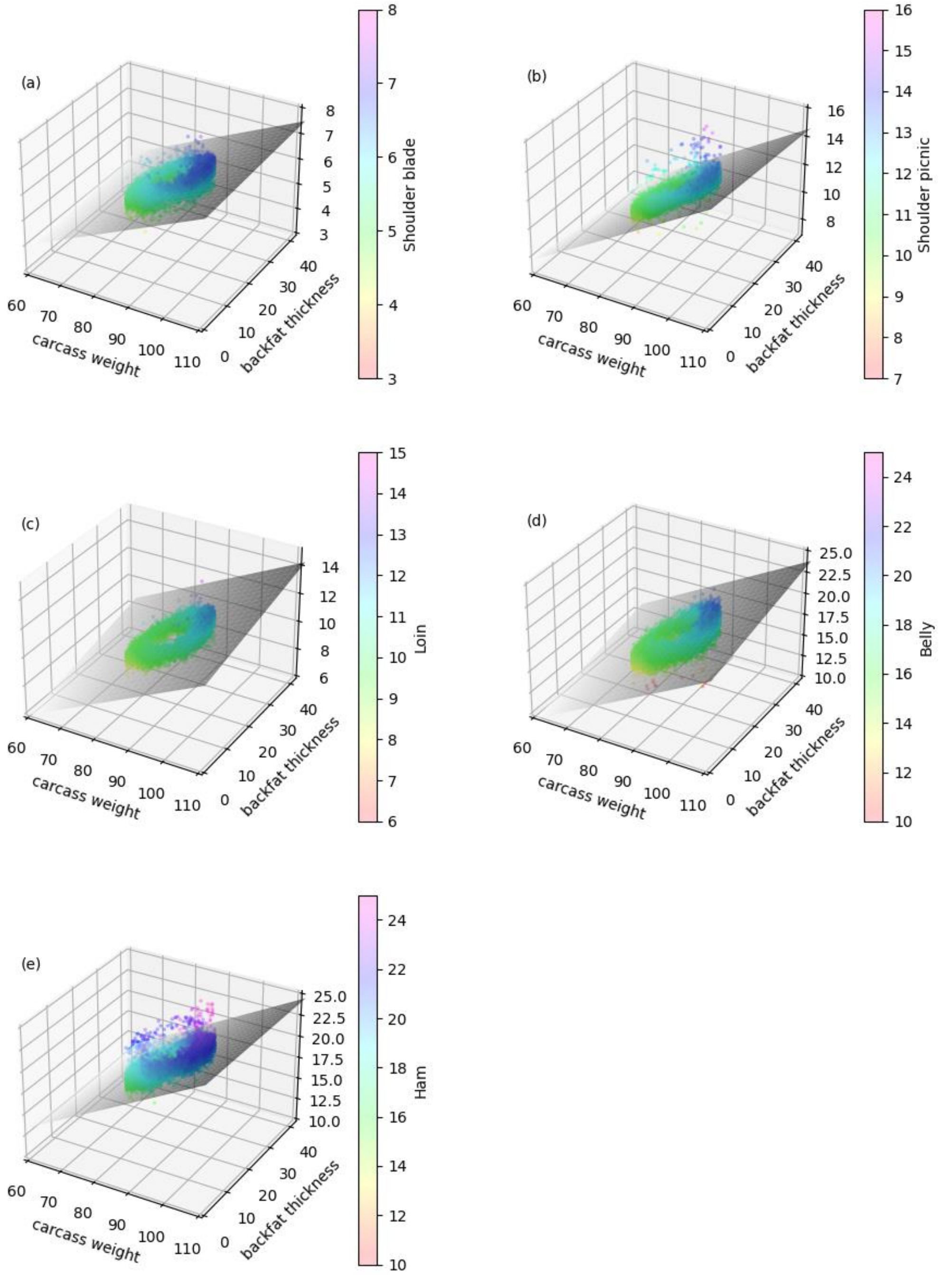

Supplementary Figure 1.3. Primal cut production (kg) as a function of carcass weight (kg) and backfat thickness (mm) for pigs in group III. The primal cut production in each graph represents (a) shoulder blade, (b) shoulder picnic, (c) loin, (d) belly, and (e) ham. The black plots in each graph are the graphs of the regression models from Table 4.3 with carcass weight and backfat thickness as independent variables and each primal cut production as the dependent variable.

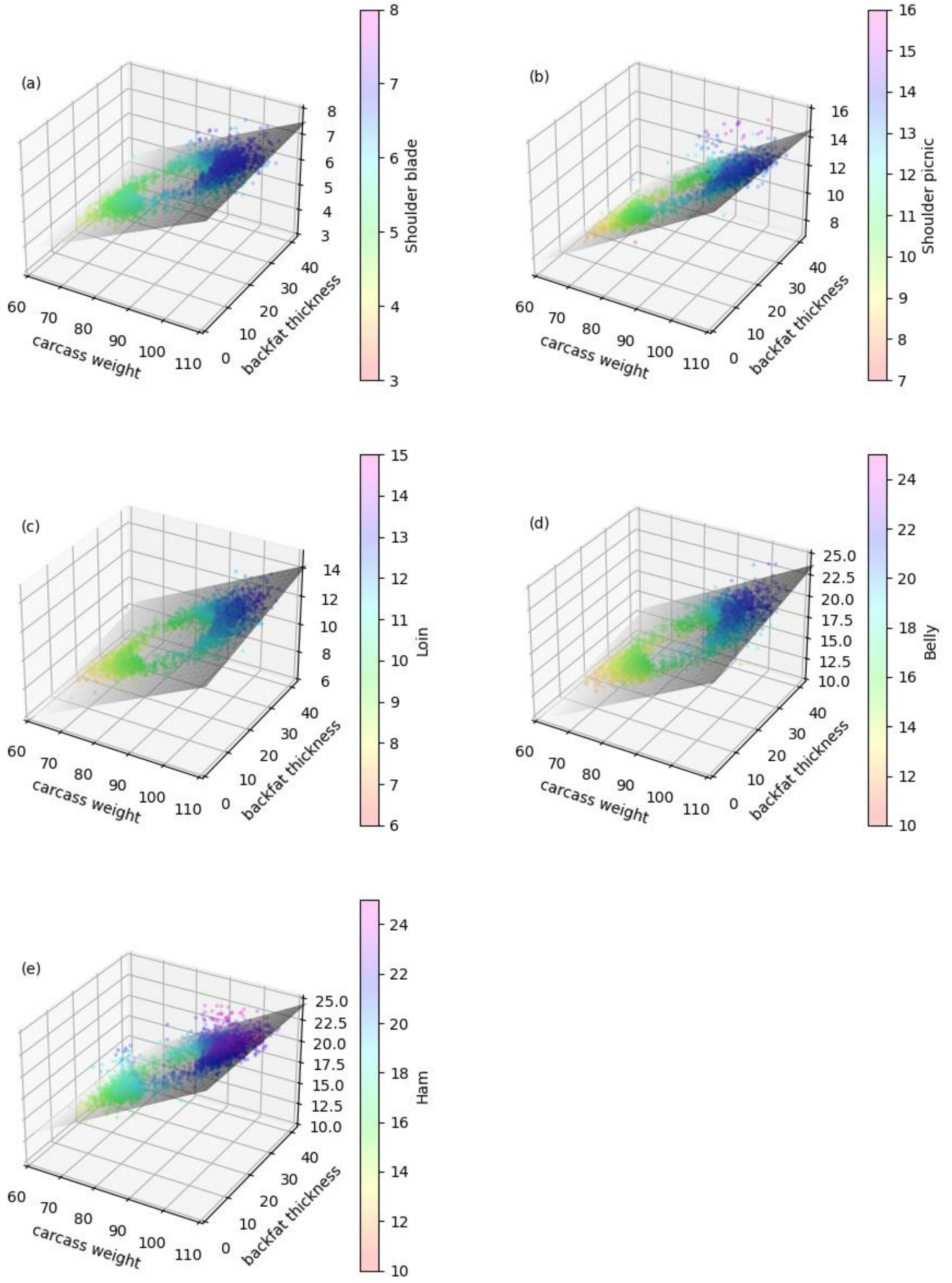

Supplementary Figure 1.4. Primal cut production (kg) as a function of carcass weight (kg) and backfat thickness (mm) for pigs in group IV. The primal cut production in each graph represents (a) shoulder blade, (b) shoulder picnic, (c) loin, (d) belly, and (e) ham. The black plots in each graph are the graphs of the regression models from Table 4.4 with carcass weight and backfat thickness as independent variables and each primal cut production as the dependent variable.
