# Supplementary material for: New management grading for pig farms: management grading system using pig carcass weight, back fat thickness and k-means algorithm
Source: Anim Biosci. 2024 Aug 26;38(2):371–80. doi: 10.5713/ab.24.0350 (PMC11725751; doi:10.5713/ab.24.0350)
Supplement: Supplementary file 2 [file ab-24-0350-Supplementary-Table.pdf]

Supplementary Tables and Figures

Supplementary Table 1. Mean and standard deviation of carcass weight and backfat thickness for pigs used in the study.

	Carcass weight (kg)	Backfat thickness (mm)
Mean	86.51	22.22
standard deviation	5.40	3.88

The number of pigs used in the study was 48,298, slaughtered in June and July.

Supplementary Table 2.1. Confidence intervals for regression equations for five primal cuts of pigs in group I

Dependent variable	confidence intervals of β0		confidence intervals of β1		confidence intervals of β2	
	0.025	0.975	0.025	0.975	0.025	0.975
Shoulder blade	0.547	0.954	0.056	0.061	-0.006	-0.000
Shoulder picnic	-0.917	-0.338	0.141	0.147	-0.032	-0.024
Loin	-1.440	-0.892	0.112	0.119	0.048	0.056
Belly	-4.021	-2.605	0.204	0.220	0.056	0.075
Ham	-0.455	0.892	0.211	0.226	-0.014	0.005

Supplementary Table 2.2. Confidence intervals for regression equations for five primal cuts of pigs in group II

Dependent variable	confidence intervals of β0		confidence intervals of β1		confidence intervals of β2	
	0.025	0.975	0.025	0.975	0.025	0.975
Shoulder blade	0.347	0.517	0.060	0.062	0.002	0.004
Shoulder picnic	-0.817	-0.565	0.141	0.144	-0.022	-0.019
Loin	-1.111	-0.878	0.113	0.115	0.048	0.052
Belly	-3.468	-2.866	0.206	0.213	0.065	0.074
Ham	0.308	0.873	0.213	0.219	-0.015	-0.008

Supplementary Table 2.3. Confidence intervals for regression equations for five primal cuts of pigs in group III

Dependent variable	confidence intervals of β0		confidence intervals of β1		confidence intervals of β2	
	0.025	0.975	0.025	0.975	0.025	0.975
Shoulder blade	0.390	0.489	0.060	0.061	0.004	0.005
Shoulder picnic	-0.720	-0.569	0.140	0.142	-0.018	-0.016
Loin	-1.089	-0.946	0.115	0.116	0.045	0.048
Belly	-3.433	-3.088	0.209	0.213	0.068	0.074
Ham	0.422	0.755	0.213	0.217	-0.013	-0.008

Supplementary Table 2.4. Confidence intervals for regression equations for five primal cuts of pigs in group IV

Dependent variable	confidence intervals of β0		confidence intervals of β1		confidence intervals of β2	
	0.025	0.975	0.025	0.975	0.025	0.975
Shoulder blade	0.246	0.352	0.060	0.062	0.005	0.007
Shoulder picnic	-0.799	-0.628	0.140	0.142	-0.015	-0.012
Loin	-1.062	-0.895	0.115	0.117	0.041	0.045
Belly	-3.244	-2.872	0.209	0.214	0.059	0.067
Ham	0.065	0.427	0.215	0.220	-0.014	-0.007

Supplementary Table 3. Centroid coordinates for each cluster

Centroid	abs z-score of carcass weight	abs z-score of backfat thickness
cluster 1	0.2690805	0.30973118
cluster 2	0.34590601	0.93179447
cluster 3	0.80756976	0.32579825
cluster 4	0.41424701	1.6461839
cluster 5	1.1533521	1.10633368
cluster 6	1.45804912	0.37097497
cluster 7	0.92357137	2.43549613
cluster 8	2.21918036	1.93376544
cluster 9	2.42865688	0.68700627

Supplementary Table 4. Regression equations for five primal cuts weight in whole data

Dependent variable	Intercept (β0)	Regression coefficient of Carcass weight (β1)	Regression coefficient of Backfat thickness (β2)	MAE	R^2
Shoulder blade	0.4115	0.0605	0.0043	0.17	0.698
Shoulder picnic	-0.6791	0.1416	-0.0172	0.24	0.838
Loin	-1.0199	0.1154	0.0467	0.24	0.841
Belly	-3.2091	0.2107	0.0684	0.59	0.730
ham	0.5539	0.216	-0.0108	0.53	0.713

The confidence intervals for each regression are shown in supplementary table 5. The form of the regression expression is as follows: $y = \beta 1 x1$(carcass weight) + $\beta 2 x2$(backfat thickness) + $\beta 0$

Supplementary Table 5. Confidence intervals for regression equations for five primal cuts of whole data

Dependent variable	confidence intervals of β0		confidence intervals of β1		confidence intervals of β2	
	0.025	0.975	0.025	0.975	0.025	0.975
Shoulder blade	0.38	0.443	0.06	0.061	0.004	0.005
Shoulder picnic	-0.726	-0.632	0.141	0.142	-0.018	-0.016
Loin	-1.064	-0.976	0.115	0.116	0.046	0.047
Belly	-3.319	-3.1	0.209	0.212	0.067	0.07
Ham	0.449	0.659	0.215	0.217	-0.013	-0.009
